# Polysaccharide Cryogels Containing β-Cyclodextrin for the Delivery of Cannabidiol

**DOI:** 10.3390/pharmaceutics13111774

**Published:** 2021-10-23

**Authors:** Denitsa Momekova, Yavor Danov, Georgi Momekov, Ervin Ivanov, Petar Petrov

**Affiliations:** 1 Faculty of Pharmacy, Medical University of Sofia, 1000 Sofia, Bulgaria; dmomekova@pharmfac.mu-sofia.bg (D.M.); gmomekov@gmail.com (G.M.); ervin.ivanov@gmail.com (E.I.); 2 Institute of Polymers, Bulgarian Academy of Sciences, 1113 Sofia, Bulgaria; yavor.danov@polymer.bas.bg; 3 PobelchGle Ltd., 1618 Sofia, Bulgaria

**Keywords:** cannabidiol, cryogels, β-cyclodextrin, 2-hydroxyethyl cellulose, drug delivery

## Abstract

Cannabidiol (CBD) has attracted increasing interest due to its therapeutic potential for treating numerous diseases. However, CBD is very lipophilic and has very unfavorable pharmacokinetics and low bioavailability. Efforts are focused on developing drug delivery systems for enhanced solubilization and therapeutic activity of CBD. Here, we report the preparation of original super-macroporous cryogels from 2-hydroxyethyl cellulose (HEC) and β-cyclodextrin (β-CD) designed for the topical delivery of CBD. The cryogels were synthesized by photochemical crosslinking in a frozen aqueous system, purified, and then loaded with CBD. The effect of HEC/β-CD mass ratio (100:0; 50:50; 40:60 and 20:80) in the reaction mixture on the reaction efficiency, physico-mechanical properties of cryogels, drug release profile, and antineoplastic potential were evaluated in detail. The cryogels showed a bi-phasic release behavior: initial burst release in the first 3 hours followed by slower drug release which can be beneficial in the treatment of cutaneous neoplastic diseases.

## 1. Introduction

The natural terpenophenolic compound cannabidiol from *Cannabis sativa* is endowed with a plethora of pharmacological activities, including analgesic, antidepressant, antiepileptic, anti-inflammatory, antioxidant, cytotoxic, and antineoplastic, among others [1,2,3,4,5,6,7,8,9]. It has been the subject of numerous experimental and clinical studies and has been granted orphan drug designation for the treatment of rare pediatric forms of epilepsy [1,6,7,8]. One of the most intriguing avenues for cannabidiol research is its evaluation as a possible antineoplastic agent based on prominent cytotoxicity in cell lines of different origins and cell types [10]. Nevertheless, the compound has inherited pharmacokinetic, pharmaceutical, and pharmacokinetic features that generally hamper its development beyond the experimental stage in this area of phytocannabinoid research [4,6,11]. This agent is very lipophilic (logP 6.3) and has very unfavorable and highly variable pharmacokinetic parameters following oral or oromucosal uptake with a bioavailability of about 6% [11]. The typical dosing regimens yield plasma concentrations within the low nanomolar range [4], whereas the documented inhibitory concentrations against tumor cell lines are largely micromolar [10]. This gap is certainly not insurmountable with conventional drug delivery platforms and dosing schedules. On these grounds, numerous drug delivery systems for cannabidiol based on nanocarriers or nanocomposite materials, such as self-emulsifying systems, nano-spheres, micelles, and inclusion complexes have been developed [11,12,13,14,15,16]. Since the oral route of administration for CBD has been associated with poor bioavailability, other more effective routes for sustained therapeutic delivery of CBD have been evaluated. In this regard, administration through the skin has been reported as a promising approach for CBD delivery, with several advantages [17,18]. It is considered a more user-friendly route that bypasses the hepatic first-pass effect and prevents the degradation of drug molecules by enzymes in the gut. Indeed, several recent studies have demonstrated that formulations (gels/creams) containing ingredients such as oils, polymers, alcohols, enhances, etc., enhance the delivery of CBD via topical/transdermal routes [19,20,21,22,23]. These systems have been assessed for pain and inflammation treatment. An area of special interest is the possibility for the topical delivery of CBD at effective concentrations to primary skin tumors or cutaneous metastases. CBD has been shown to effectively inhibit cell lines from cutaneous T-cell lymphoma (CTCL) which forms a rationale for its development as a topical therapeutic modality for this neglected neoplastic disease [24]. However, there is limited literature available about the topical/transdermal delivery of CBD intended for skin cancer treatment. Very recently, we have reported on a nanocomposite system based on CBD-loaded polymeric micelles, embedded in a hydroxyethyl cellulose cryogel matrix [15]. Such cryogel-based drug delivery systems are considered beneficial for the sustained local delivery of CBD to patients with CTCL. Nonetheless, at lower drug concentrations, the cytotoxic effect of the free CBD was more pronounced. Therefore, systems with bi-phasic release behavior (combination of burst and sustained release) might offer new perspectives for efficient therapy, providing prompt exposure of malignant cells to the initially released CBD and sustained inhibitory activity, afforded by the second drug release phase. In this vein, the present contribution describes a novel drug delivery platform for the topical delivery of cannabidiol, based on CBD/β-cyclodextrin inclusion complexes within biocompatible cryogels. The cryogels were synthesized through the photochemical crosslinking of HEC and β-CD-based crosslinking agents in a frozen aqueous system and then loaded with CBD. The effect of the HEC/β-CD mass ratio on the swelling behavior and mechanical properties of cryogels, drug loading and release profile, and antineoplastic potential was evaluated.

## 2. Materials and Methods

### 2.1. Materials

2-Hydroxyethyl cellulose (1.3 kDa) was donated by Hercules Inc. Aqualon Division (Wilmington, DE, USA). β-Cyclodextrin, acryloyl chloride, trimethylamine (TEA), (4-benzoylbenzyl) trimethylammonium chloride (BBTMAC), 1,4-dioxane, dimethyl sulfoxide (DMSO), dimethylformamide (DMF), ethanol, RPMI growth medium, and fetal bovine serum were purchased from Sigma-Aldrich (Darmstadt, Germany). Cannabidiol was kindly donated by PBG GLOBAL LTD., Sofia, Bulgaria. All materials were used as received.

### 2.2. Methods

#### 2.2.1. Synthesis of β-CD-Acrylate 

The crosslinking agent was synthesized by following a procedure described elsewhere [25,26]. Briefly, β-CD (2.00 g, 1.76 mmol) was dissolved in dimethylformamide (15 mL) in a round-bottomed flask. Then, triethylamine (1.71 mL, 12.32 mmol) was added. The flask was placed in an ice bath to cool down the mixture at 0 °C, and after that, acryloyl chloride (1 mL, 12.32 mmol) was added dropwise. The reaction was carried out for 16 h at 20 °C with intensive stirring (700 rpm). The insoluble white salt was removed by filtration and the residual solution was concentrated by rotary evaporation and precipitated in 10-fold excess of cold acetone −20 °C). The final product, β-CDAc_3_, was recovered by filtration and dried. The yield was equal to 61%. 

#### 2.2.2. Synthesis of Cryogels 

Cryogels were fabricated by the photo-crosslinking of HEC and β-CDAc_3_ in a frozen aqueous system [25]. Initially, HEC (0.1 g) was dissolved in deionized water (6 mL) under stirring at 40 °C for 30 min and kept overnight at room temperature. Given amounts of β-CDAc_3_ (0.1, 0.15, and 0.4 g) and BBTMAC (0.01 g, 10 mass % to HEC) were dissolved in deionized water (4 mL) under stirring at room temperature and then added to the aqueous solution of HEC. Ten portions of 1 mL were distributed into Teflon dishes (2 cm in diameter) to form a ca.0.5 cm thick layer, and the dishes were placed in a freezer at −20 °C for 2 h. Finally, the frozen aqueous system was irradiated with the full spectrum of UV–Vis light with a “Dymax 5000-EC” UV equipment with a 400 W metal halide flood lamp for 5 min (dose rate = 5.7 J cm^−2^ min^−1^).

#### 2.2.3. Calculation of Gel Fraction Yield and Swelling Degree

All samples, after UV irradiation, were purified by extraction in distilled water for 7 days at room temperature, freeze-dried, and weighed. The gel fraction yield and swelling degree of the cryogels were determined gravimetrically using the following equations:

(1) $$GF\left(\%\right)=\frac{\mathrm{mass}\;\mathrm{of}\;\mathrm{freeze}\;\mathrm{dried}\;\mathrm{cryogel}}{\mathrm{initial}\;\mathrm{mass}\;\mathrm{of}\;\mathrm{HEC}\;\mathrm{and}\;\beta -\mathrm{CDAc}}\times 100$$

(2) $$SD=\frac{\mathrm{mass}\;\mathrm{of}\;\mathrm{swollen}\;\mathrm{cryogel}}{\mathrm{mass}\;\mathrm{of}\;\mathrm{freeze}\;\mathrm{dried}\;\mathrm{cryogel}}$$

#### 2.2.4. Calculation of β-CD Fraction Incorporated in Cryogel Network

Cryogel disks were immersed in 10 mL of Tris HCl buffer (50 mM, pH = 8.93) containing phenolphthalein (0.25 mmol L^−1^) for 15 min at 25 °C and then removed. The absorbance of the solution was measured at 550 nm. The amount of β-CD incorporated in the cryogel network was calculated with the aid of the calibration curve of phenolphthalein in the Tris HCl buffer in the concentration range of 0.05 to 0.25 mmol L^−1^ (y = 8919.8x − 0.28457, R^2^ = 0.9867).

#### 2.2.5. Loading of Cannabidiol in Cryogels

Freeze-dried pure HEC or HEC/β-CD cryogels were placed into Teflon dishes and then 1 mL of CBD was dissolved in dioxane (0.5 g L^−1^) and was added to each sample (0.5 mg of CBD in each gel). The samples were kept in the dark for 24 h before adding 0.2 mL of distilled water. The two solvents were then removed by freeze-drying to produce CBD-loaded cryogels.

The loading efficacy was evaluated by immersing the disks in 2 mL of water to swell for 10 min. Afterward, 8 mL of ethanol (96%) was added and disks were kept for 1 h at room temperature. Then, the amount of extracted CBD was determined spectrophotometrically at λ = 237 using the calibration curve of CBD in ethanol in the concentration diapason of 0.002 to 0.1 g L^−1^ (y = 12.13751x − 0.11934, R^2^ = 0.9992).

#### 2.2.6. Cannabidiol Release 

The CBD released from pure HEC and HEC/β-CD cryogels was evaluated for a period of 24 h at 35 °C. The dissolution tests were performed in 50 mL acetate buffer (pH 5.5) under constant stirring at 50 rpm. At fixed time intervals, 2 mL of the release medium was taken and the amount of released CBD was evaluated spectrophotometrically at λ = 237 nm. The percentage of drug release was calculated based on a standard curve of CBD found in the dissolution medium in the concentration range from 0.002 to 0.1 g.L^−1^ (y = 20.33498.x + 0.00718 R^2^ = 0.997).

#### 2.2.7. Cell lines and Culture Conditions

The cytotoxic activity of CBD and its formulations was assessed against two cell lines: MJ cells (CTCL, Mycosis fungoides) and HUT-78 cells (cutaneous T-cell lymphoma, CTCL- Sézary syndrome). The cell lines were purchased from the German Collection of Microorganisms and Cell Cultures (DSMZGmbH, Braunschweig, Germany). The growth medium was 90% RPMI-1640 + 10% FBS. The cells were grown in a controlled environment—cell culture flasks at 37 °C in an incubator ‘BB 16-FunctionLine’ Heraeus (Kendro, Hanau, Germany) with a humidified atmosphere and 5% CO_2_.

#### 2.2.8. Cytotoxicity Assessment

The cytotoxicity of CBD-loaded HEC and HEC/β-CD cryogels was evaluated in a comparative way vs. the free CBD through MTT-dye reduction assay against two human tumor cell lines: HUT-78 (cutaneous T-cell lymphoma, CTCL-Cesary syndrome) and MJ (CTCL-Mycosis fungoides). Briefly, exponentially growing cells were plated in 6-well flat-bottomed plates (3 mL/well) at a density of 1 × 10^5^/mL, and after 24 h incubation at 37 °C, cells were treated with various fragments of the tested CBD-loaded macroporous disks, corresponding to CBD concentrations of 0.01 to 0.08 mg/ml for 72 h. At least 3 wells were used for each concentration. After the exposure period, 300 μL of MTT solution (10 g L^−1^ in PBS) was added to each well. Afterwards, the samples were incubated for 4 h at 37 °C and the MTT–formazan crystals that had formed were then dissolved by adding 5% formic acid-acidified 2-propanol. The MTT–formazan absorption was recorded using a LabeximLMR-1 microplate reader at 580 nm. Cell survival fractions were calculated as the percentage of the untreated control. In addition, IC_50_ values were derived from the concentration–response curves.

#### 2.2.9. Proton NMR Analyses

The proton NMR spectrum of β-CDAc_3_ was recorded using a 600 MHz Bruker AC spectrometer in D_2_O.

#### 2.2.10. Freeze-Drying

The materials were freeze-dried in an Alpha1-2 freeze dryer (Martin Christ) for 24 h at 0.02 mbar and −55 °C.

#### 2.2.11. Rheological Measurements

Dynamic rheological measurements were performed using a HaakeRheoStress 600 rheometer with a parallel plate sensor system (20 mm diameter) and Peltier temperature controller. Dynamic storage (G′) and loss (G′′) moduli were measured at 25 °C, in the 0.03–10 Hz frequency range in controlled deformation mode at γ = 0.005, which is inside the linear viscoelastic regime. Three runs of each sample were performed.

#### 2.2.12. Scanning Electron Microscopy

Scanning electron microscopy (SEM) was conducted by using a JEOL 6390 instrument with an accelerating potential of 8.00 kV. All freeze-dried specimens were fractured and fixed on a glass substrate with a nail polish, and coated with gold for 60 s.

#### 2.2.13. Differential Scanning Calorimetry 

Differential scanning calorimetry (DSC) was performed on a Perkin Elmer DSC-8500 apparatus. Samples with room humidity were tested from 30 to 120 °C with a 10 °C/min heating rate under nitrogen flow (50 mL/min).

#### 2.2.14. Data Processing and Statistics

The MTT-bioassay data are representative of six independent experiments. The cell survival data were normalized as a percentage of the solvent-treated control (set as 100% viability) and fitted to sigmoidal dose-response curves. The corresponding IC_50_ values (concentrations causing 50% suppression of cellular viability) were calculated by non-linear regression analysis using GraphPad Prizm Software for PC.

The statistical processing of biological data included the paired Student’s t-test whereby values of *p* ≤ 0.05 were considered as statistically significant.

## 3. Results

### 3.1. Synthesis and Characterization of Cryogels

**Figure 1:**
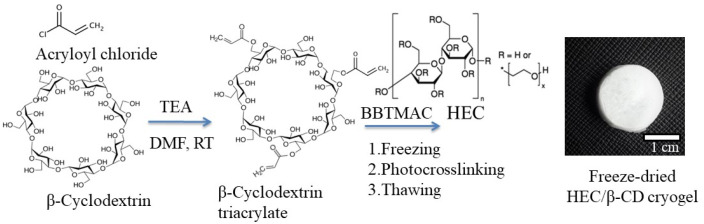
Synthesis of HEC/β-CD cryogel via cryogenic treatment and photochemical crosslinking in a frozen state.

Cryogels of different compositions were synthesized by photochemical crosslinking of the biocompatible and biodegradable polysaccharide HEC and modified oligosaccharide β-CD (100:0, 50:50; 40:60 and 20:80 mass ratio) in a frozen aqueous system (Figure 1). The water-soluble BBTMAC was used as a photoinitiator.

**Figure 2:**
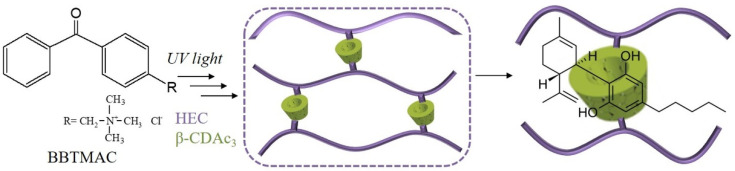
Formation of HEC/β-CD polymer network and “host-guest” complex between β-CD and CBD.

Firstly, the oligosaccharide β-CD was transformed into a crosslinking agent bearing acrylate groups via reaction with acryloyl chloride in the presence of triethylamine as described elsewhere [25,26]. To obtain a multifunctional crosslinking agent, i.e., several acrylate groups per one β-CD molecule, a given excess of acryloyl chloride was used. The ^1^H-NMR analysis of the isolated reaction product revealed a degree of substitution (DS) of about three. The calculation was made considering the relative peak integrals assigned to the vinyl protons of 5.8–6.5 ppm and the β-CD protons at 5.0 ppm (Figure S1). Next, the super-macroporous cryogels were synthesized by a protocol including the cryogenic treatment (freezing for 2 h) of aqueous solution of HEC, β-CDAc_3_ and BBTMAC, and UV irradiation for 5 min. Figure 2 schematically represents the formation of a three-dimensional HEC/β-CD polymer network designed for “host-guest” complexation between CBD and β-CD inner cavity. We assumed that HEC and β-CDAc_3_ were incorporated into the polymer network equivalently to their initial mass ratios. This was confirmed by experiments based on the inclusion of phenolphthalein in the β-CD cavities of cryogels. Considering that the inclusion complex β-CD/phenolphthalein follows the 1:1 stoichiometry [27], the calculated fraction of β-CD in cryogels was identical to the fraction in the reaction mixture.

**Figure 3:**
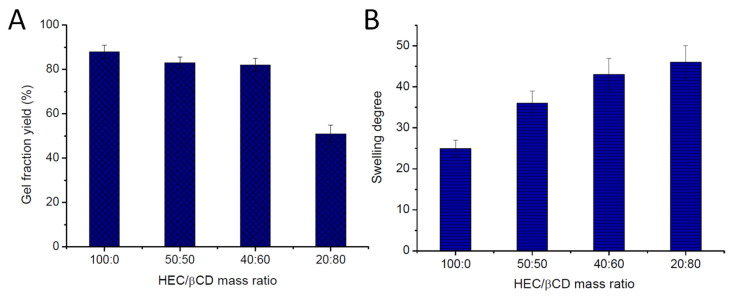
Effect of HEC/β-CDAc_3_ mass ratio on the gel fraction yield (**A**) and swelling degree of cryogels (**B**).

Figure 3 shows the effect of the HEC/β-CDAc_3_ mass ratio on the GF yield and swelling degree of cryogels. High GF yield values (82–88% ± 3%) were calculated for all gels, except the sample prepared from the HEC/β-CDAc_3_ (20:80) mixture. The GF yield of HEC/β-CDAc_3_ (50:50 and 40:60) was identical to the GF yield of pure HEC cryogel, suggesting a high degree of incorporation of both reagents into the polymer network. An inverse dependence of SD on the GF yield was observed. The pure HEC gel absorbed significantly less water (SD 25 ± 2) than HEC/β-CD cryogels (SD 36– 46 ± 4). The lower the HEC fraction, the higher the swelling degree of gels (Figure 3B).

**Figure 4:**
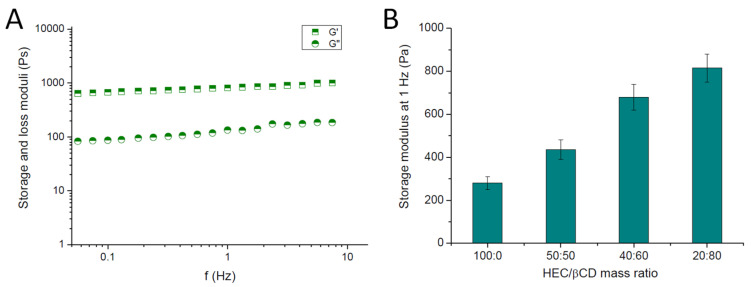
Storage (G′) and loss (G′′) moduli of HEC/β-CD (2:8) cryogels as a function of frequency (**A**), and effect of HEC/β-CD mass ratio on the storage modulus of cryogels (**B**).

The viscoelastic properties of HEC/β-CD cryogels were assessed by dynamic rheological measurements. All studied materials exhibited the typical behavior for gels—the storage and loss moduli were frequency independent, and no crossover was registered in the 0.03 to 10 Hz range. As illustrated in Figure 4A, G′ is nearly one order of magnitude higher than G′′, telling us that the elastic response of the material to the applied shear stress significantly exceeds the viscous response. On the other hand, G′ raised proportionally with the increase in β-CD fraction into the polymer network (Figure 4B). Thus, an obvious reinforcing effect of β-CD (approximately 2.5 times) was found as compared to the pure HEC and HEC nanocomposite cryogels, containing polymer micelles [15].

### 3.2. Drug Loading and Release

**Figure 5:**
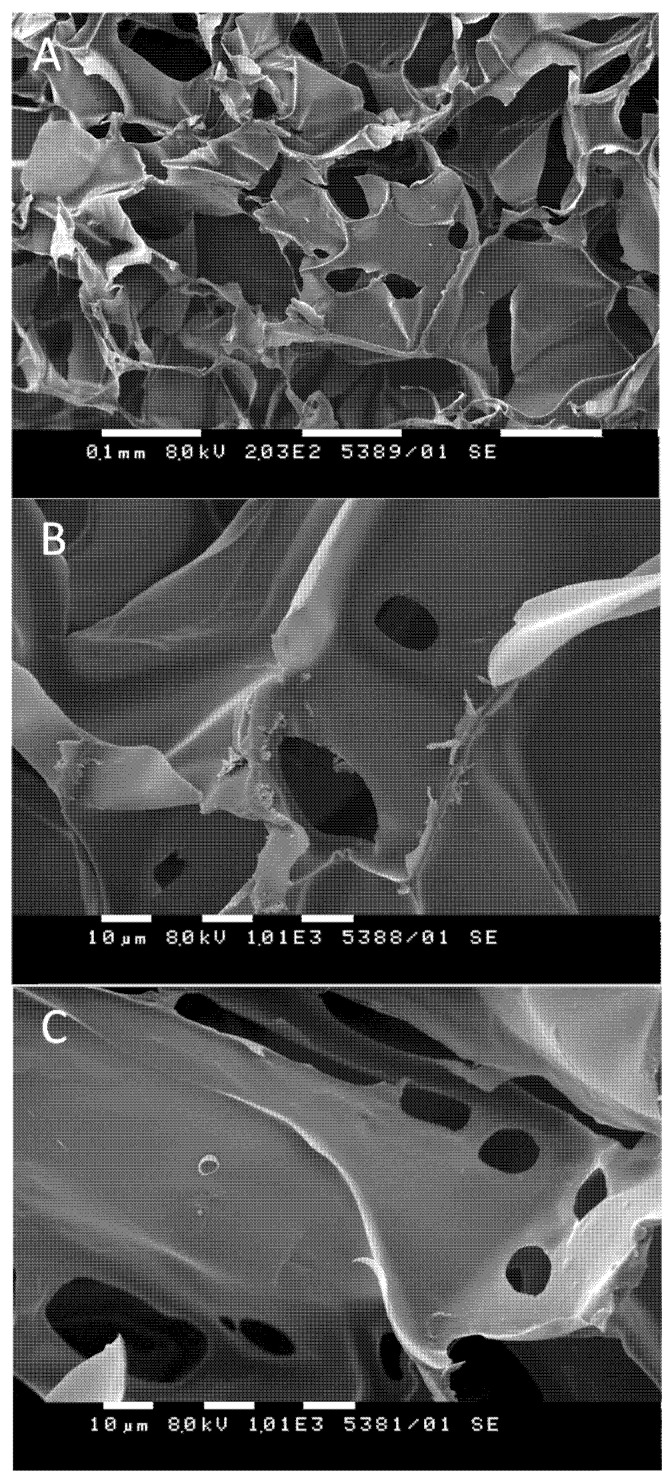
Selected SEM micrographs of CBD-loaded (**A**,**B**) and pure (**C**) HEC/β-CD cryogels (20:80).

The macroporous structure of the elaborated HEC/β-CD cryogels promoted a high loading efficacy. The loading procedure involves soaking of the dried cryogel matrices in a 1,4-dioxane (or DMSO) solution of CBD (0.5 g L^−1^), 24 h of incubation, and adding a given amount of water to trigger the formation of an inclusion complex between CBD molecules and the interior cavities of β-CD incorporated in the polymer matrix. Next, the solvents were removed by freeze-drying to yield a 100% loading efficacy for all tested formulations. Scanning electron microscopy analysis of selected samples revealed the existence of small particles randomly adsorbed on the inner cryogel surface (Figure 5A,B). One can speculate that these particles originate from CBD since the surface of the pure cryogel seems smooth (Figure 5C). However, SEM is not a quantitative method, and, based on such observation, it is not reliable to quantify the CBD fractions embedded in β-CD cavities nor adsorbed on the surface of the polymer matrix. 

**Figure 6:**
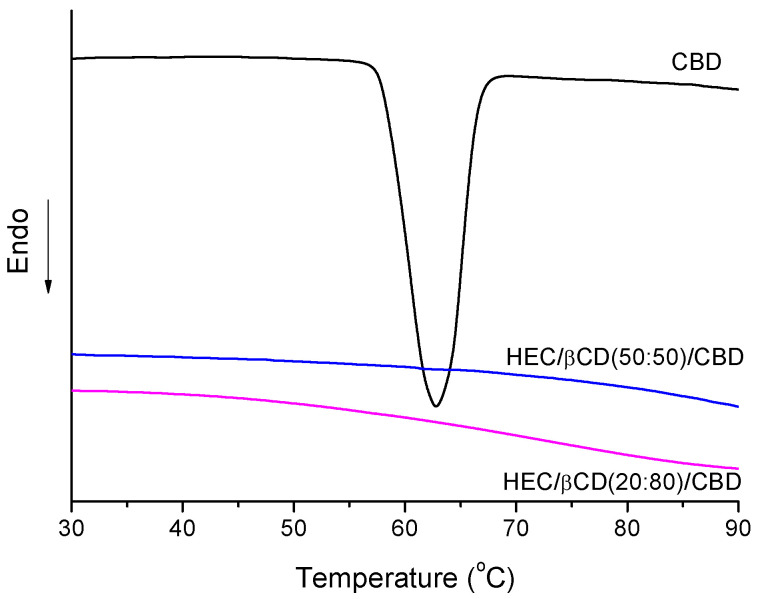
DSC thermograms of CBD-loaded HEC/β-CD cryogels and pure CBD.

DSC measurements provided additional information about the phase state of CBD loaded in HEC/β-CD carriers. Melting curves of pure CBD and HEC/β-CD cryogel of two different compositions are shown in Figure 6. The pure CBD is a crystalline white solid which exhibited a melting peak at 63 °C. This peak was not detected for the CBD-loaded cryogel samples, suggesting that CBD is in an amorphous state probably due to the loading procedure and/or some specific interaction with the polymer matrix.

**Figure 7:**
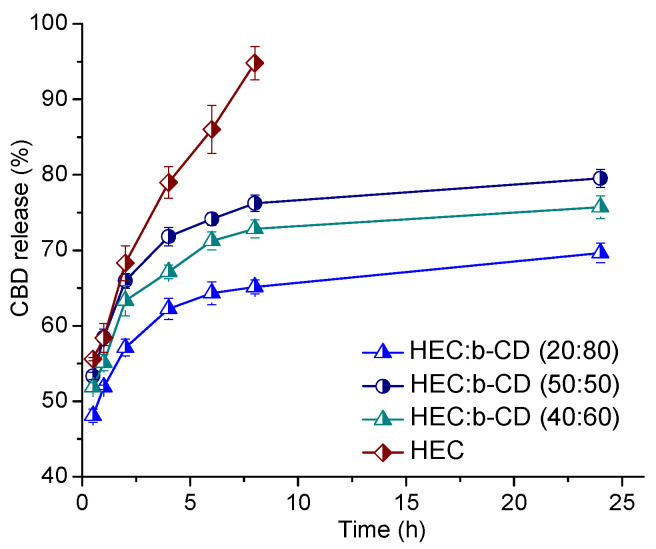
In vitro drug release of cannabidiol-loaded pure HEC and HEC/β-CD macroporous cryogel carriers in acetate buffer (pH = 5.5) at 35 °C. Mean values ± SD (*n* = 3).

A rough idea about the CBD fraction, included in the β-CD cavities, can be attained from in vitro drug release experiments. The release profiles of cannabidiol from hydrogel matrixes of different compositions (HEC/β-CD 100:0, 50:50, 40:60, and 20:80) were studied and the corresponding graphs are presented in Figure 7. The release studies were conducted in an acetate buffer solution (pH 5.5) at 35 ± 0.5 °C, in accordance with the potential dermal route of application of the elaborated cryogel systems. The pure HEC cryogel is characterized by rapid drug release—about 96% of cannabidiol was released within the first 6 hours of the experiment. In this sample, CBD is not involved in complex with β-CD. In contrast, the HEC/β-CD cryogels quickly released about 50% of the loaded CBD, followed by a significantly slower release of another portion of CBD. In fact, for the period of 24 h, less than 80%, 70%, and 63% of the total amount of CBD was released from the carriers comprising 50%, 60%, and 80% of β-CD moiety, respectively. The higher the content of β-CD, the lower the percentage of released CBD from the matrix. From the obtained results, we can conclude that β-CD-modified polysaccharide matrixes may serve as feasible carriers providing high entrapment efficiency and bi-phasic drug release for the hydrophobic drug CBD.

### 3.3. Cytotoxicity Study

**Figure 8:**
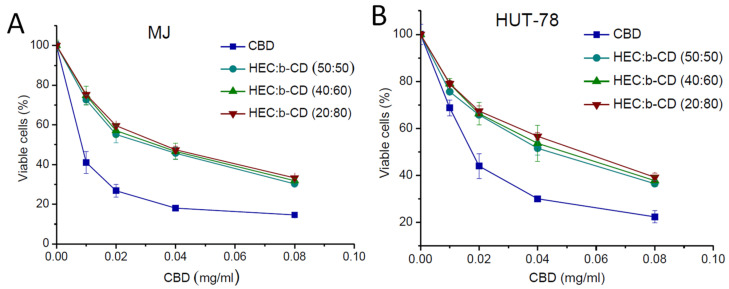
Concentration–response curves for two human tumor cell lines -MJ (A) and HUT-78 (B), determined by the MTT assay after 72 hours of continuous exposure. Each data point represents an average arithmetic value ± standard deviation of 8 independent experiments.

The cell growth inhibitory effect of CBD-loaded HEC/β-CD cryogels was investigated in a comparative fashion vs. free CBD against two human tumor cell lines -MJ (CTCL-Mycosis fungoides) and HUT-78 (cutaneous T-cell lymphoma). A standard MTT test, based on the enzymatic reduction of the yellow tetrazolium salt MTT to a violet MTT–formazan by the mitochondrial succinate dehydrogenase of vital cells, was used with minor modifications [28]. The concentration–response curves are shown in Figure 8 and the half-inhibitory concentrations (IC_50_) calculated thereof are presented in Table 1. The presented data indicate that free CBD (applied as ethanol solution) and the formulated CBD showed concentration-dependent cytotoxicity, but the anti-proliferative effect of the free drug is higher as compared to formulated CBD. Thus, IC_50_ values of the β-CD-containing cryogels were slightly higher than those of the free drug (with an identical magnitude of modulation) in both cell lines.

## 4. Discussion

Polysaccharide cryogels comprising β-cyclodextrin units were designed for solubilization and delivery of the poorly water-soluble bioactive substance cannabidiol. The study focused on fabricating cryogels comprising relatively high β-CD fraction because this is a prerequisite for complexation with more CBD molecules per unit mass of the carrier. A previous study revealed that intact cryogel from pure β-CDAc_3_ cannot be formed [26]. This is attributed to the specific feature of the β-CDAc_3_ molecule, which is bulky and inflexible. In the present case, HEC macrochains facilitated the intrachain crosslinking and thus contributed to an increase in the reaction efficiency and GF yield, respectively. The cryogels were fabricated by photochemical crosslinking in a frozen aqueous solution of HEC and β-CDAc_3._ The cryogenic treatment of aqueous solution (freezing for 2 h at −20 °C) occurred in two phases—a solid phase of pure ice crystals and a liquid microphase, containing HEC, β-CDAc_3,_ and the photoinitiator BBTMAC. Hence, the polymer network was formed in the confined space of the liquid microphase (constructing cryogel walls), and the ice crystals shaped the cryogel pores. Upon UV irradiation, BBTMAC underwent several photophysical processes affording (macro)radicals by the proton abstraction mechanism. These reactive species recombined to form a three-dimensional HEC/β-CD polymer network. Based on our previous experience, 5 min irradiation with UV light was considered an optimal time to complete the crosslinking process [25,26]. Despite their different GF yield, cryogels were intact and preserved the initial shape and size when placed in water. The crosslinking reaction can be considered highly efficient (except HEC/ β-CDAc_3_ 20:80), i.e., most of the initial polymer and crosslinking agents were incorporated into the network. The higher GF yield is associated with a rather dense polymer network, which influenced to some extent the swelling ability of gels. Initially, the freeze-dried cryogel material uptake quickly absorbed a significant amount of water (so-called free water) within cryogel pores, but the polymer network (cryogel walls) adsorbed an additional amount of bound water via hydrogen bonding. We believe that the composition (hydrophilicity) of polymer networks affected SD of gels along with the pore volume and density of the network. The incorporation of β-CD has a strong reinforcing effect on the mechanical properties of cryogel which is an advantage regarding their use for topical delivery.

A major problem associated with the loading of low molecular hydrophobic drugs in hydrogels is the difficulty associated with controlling their release, due to the small molecule’s size, as well their low affinity towards the hydrophilic polymer forming the matrix [29,30]. In this regard, our main hypothesis is that the appropriate modification of HEC matrix with β-cyclodextrin could provide a controlled release of cannabidiol. CBD was loaded by a method providing extremely high entrapment efficiency (100%). However, an issue regarding the amount of CBD included in β-CD inner cavities via the “host-guest” complex appeared. The observed differences in the release profiles of pure HEC matrix and β-CD-modified systems may be explained by the difference in the localization of cannabidiol molecules inside the macroporous cryogels. In the case of pure HEC, CBD is adsorbed into the cryogel walls, whereas in the β-CD-modified carriers, a fraction of the CBD (47–52%) is embedded inside the β-CD cavities in the form of the inclusion “host-guest” complex. CBD spontaneously forms an inclusion complex with β-CD at a 1:1 stoichiometric ratio; this process is endothermic, and the apparent stability constants of the complex are relatively high (Ks = 315) [12]. Hence, a higher β-CD concentration leads to an increased fraction of tightly bounded cannabidiol. This fact explains the observed sustained CBD release from β-CD-containing cryogel matrices. Overall, the combination of embedded (in the cavities) and adsorbed (on the inner surface) CBD afforded a bi-phasic release behavior—initial burst release in the first 3 hours followed by slower drug release. Such behavior can be beneficial in the treatment of cutaneous neoplastic diseases and, unlike the systems exhibiting delayed-release only [15], can provide prompt exposure of malignant cells to the initially released CBD and sustained inhibitory activity. Both free CBD and formulated CBD exhibited concentration-dependent cytotoxicity. The shift of loaded cannabidiol dose–response curves to higher concentrations is most probably due to the delayed release of the drug from the matrices and the fact that the treated cells were exposed to lower concentrations of the drug for the tested period compared to the free CBD. In addition, the free CBD was applied as an ethanol solution, whereas the formulated one was applied in a gel form. It should be mentioned that the absence of cytotoxicity of blank cryogel systems based on HEC was demonstrated in our previous study [15]. Thus, considering the biocompatibility, high loading efficiency, bi-phasic release profile of cannabidiol from the developed macroporous cryogel matrices, and the retained biological activity of the cargo, it can be suggested that the newly developed system is promising for dermal application in various skin malignancies.

## 5. Conclusions

Novel β-CD-containing polysaccharide cryogel carriers of cannabidiol were developed by photochemical crosslinking of HEC and β-CDAc_3_ in frozen aqueous solution. The freezing procedure enabled the fabrication of sponge-like materials characterized by large, interconnected pores. The elastic modulus of HEC/β-CD cryogels was notably higher than the G’ of pure HEC cryogel, suggesting improved mechanical properties of the novel carriers. HEC/β-CD cryogel matrices were loaded with CBD via a procedure allowing 100% drug loading efficiency. A portion of CBD formed an inclusion complex between the hydrophobic cavity of β-CD rings and drug molecules. The release profile of CBD from HEC/β-CD cryogels in slightly acidic (pH 5.5) media was notably affected by the cryogel composition. The higher the content of β-CD, the lower the percentage of released CBD from the matrix in the studied time interval of 24 h. CBD-loaded cryogels exhibited concentration-dependent cytotoxicity against two human tumor cell lines—MJ and HUT-78. Considering the good mechanical properties, high loading efficiency, and bi-phasic release behavior, initial burst release in the first 3 hours followed by slower drug release, we can conclude that the developed systems are promising candidates for dermal application in various skin malignancies.

## Figures and Tables

**Table 1:** Equieffective concentrations (IC_50_) of free and loaded CBD.

	Cell Line	MJ	HUT-78
Sample			
		IC_50_(mg/ml)	
CBD		0.010 ± 0.003	0.022 ± 0.004
CBD-HEC/β-CD (50:50)		0.030 ± 0.004	0.040 ± 0.006
CBD-HEC/β-CD (40:60)		0.033 ± 0.004	0.050 ± 0.004
CBD-HEC/β-CD (20:80)		0.040 ± 0.008	0.055 ± 0.007

## Data Availability

Not applicable.

## Supplementary Materials

The following are available online at https://www.mdpi.com/article/10.3390/pharmaceutics13111774/s1, Figure S1: Proton NMR spectrum of β-CDAc_3_.
