# Supplementary material for: Polysaccharide Cryogels Containing β-Cyclodextrin for the Delivery of Cannabidiol
Source: Pharmaceutics. 2021 Oct 23;13(11):1774. doi: 10.3390/pharmaceutics13111774 (PMC8618209; doi:10.3390/pharmaceutics13111774)
Supplement: Supplementary file 1 [file pharmaceutics-13-01774-s001.zip › pharmaceutics-1409866-supplementary.pdf]

pharmaceutics

MDPI

Supplementary Materials: Polysaccharide Cryogels Containing β-Cyclodextrin for Delivery of Cannabidiol

Denitsa Momekova, Yavor Danov, Georgi Momekov, Ervin Ivanov and Petar Petrov

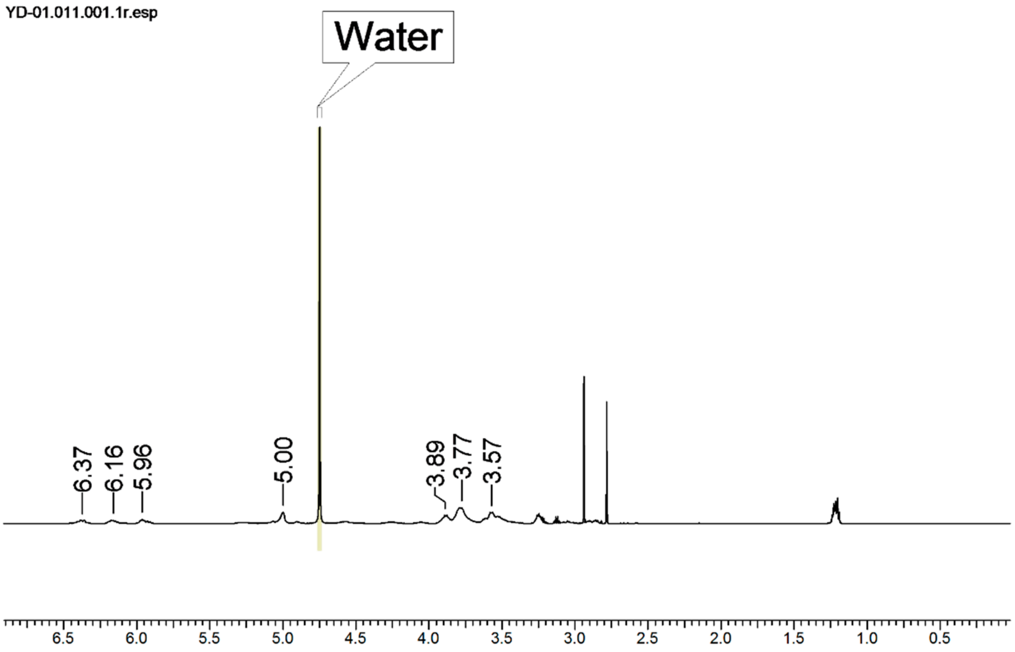

Figure S1. Proton NMR spectrum of β-CDAc in D_2O.
